# Moving a youth trauma support group online: participatory adaption, usability and pilot test: Georgina Warner

**DOI:** 10.1093/eurpub/ckac131.174

**Published:** 2022-10-25

**Authors:** A Perez Aronsson, M Thell, E Lampa, S Gupta Löfving, A Tokes, N Torakai, K Ibrahim, R Aljeshy, G Warner

**Affiliations:** 1 Department of Public Health and Caring Sciences, Uppsala University, Uppsala, Sweden; 2 Uppsala University, Uppsala

## Abstract

**Background:**

Posttraumatic stress poses a significant threat to a young person’s development. Internet-based delivery could ameliorate barriers to care, but has mostly been tested with adults. This project aimed to (i) adapt the group intervention Teaching Recovery Techniques for online delivery through a participatory process, (ii) investigate the usability of the online format and (iii) pilot the new format.

**Methods:**

Adaption recommendations were generated through participatory workshops with service users and providers, and consultation with an advisory panel with professionals and parents. Usability testing was conducted with intervention leaders (n = 5) and youth (n = 5). The public involvement in the project was assessed through a multi-method approach including behavioural observations, questionnaires and field notes. A pilot study (n = 14) is ongoing.

**Results:**

The workshops focused on safety, participation and learning. Recommendations included an emergency response protocol, communication strategies, and guidance on intervention delivery. Whilst the advisory panel largely agreed, points of disagreement included workshop ideas around personalisation, where the panel conveyed the importance of consistency in manualised interventions. Usability testing highlighted the need for explicit guidance, particularly on safety processes.

**Conclusions:**

Online delivery of trauma group support requires adaptions to ensure positive group dynamics, learning and safety. Yet, some adaptions resulting from the usability testing were also relevant to the original format, pointing to the need for more extensive use of usability testing across intervention manuals. The young people, parents and professionals involved in the project provided rich and varied perspectives, illustrating the value of broad stakeholder engagement. The ongoing pilot study explores the feasibility of online delivery, including youth perceptions of the format.

**Key messages:**

• The varied perspectives in the participatory process highlighted the importance of broad stakeholder engagement for interventions to be equally evidence-based and adapted to the target population.

• The current pilot study explores the feasibility of online delivery, including youth perceptions of the format, in order to assess the potential for scale up.

